# Heart ABCA1 and PPAR- α Genes Expression Responses in Male rats: Effects of High Intensity Treadmill Running Training and Aqueous Extraction of Black Crataegus-Pentaegyna

**DOI:** 10.5812/cardiovascmed.13892

**Published:** 2013-10-28

**Authors:** Abbass Ghanbari-Niaki, Safieyh Ghanbari-Abarghooi, Fatemeh Rahbarizadeh, Navabeh Zare-Kookandeh, Monireh Gholizadeh, Fatemeh Roudbari, Asghar Zare-Kookandeh

**Affiliations:** 1 Exercise Biochemistry Branch, Faculty of Physical Education and Sports Science, University of Mazandaran, Babolsar, IR Iran; 2 Department of Medical Biotechnology, School of Medical Sciences, University of Tarbiat Modarres, Tehran, IR Iran; 3 Department of Molecular and Cell Biology, University of Mazandaran, Babolsar, IR Iran; 4 Tehran Heart Center, Tehran University of Medical Sciences, Tehran, IR Iran

**Keywords:** Heart, PPAR-α, Running, Crataegus Extract, Rats

## Abstract

**Introduction:**

Heart as a high metabolic and aerobic tissue is consuming lipid as a fuel for its energy provision at rest during light and moderate exercise, except when lactate level is higher in blood circulation. It has been shown that any type of regular exercise and crataegus species would improve cardiovascular function and minimizes several risk factors via stimulating lipid metabolism by acting on enzymes and genes expression such as ABCA1 and PPAR α which are involving in this process.

**Materials and Methods:**

Twenty Wistar male rats (4-6 weeks old, 140-173 g weight) were used. Animals were randomly classified into training (n = 10) and control (n = 10) groups and then divided into saline-control (SC), saline-training (ST), Crataegus-Pentaegyna -control (CPC), and Crataegus-Pentaegyna -training (CPT) groups. Training groups have performed a high-intensity running program (at 34 m/min (0% grade), 60 min/day, 5 days/week) on a motor-driven treadmill for eight weeks. Animals were orally fed with Crataegus-Pentaegyna extraction (500mg/kg) and saline solution for six weeks. Seventy- two hours after the last training session, rats were sacrificed, hearts were excised, cleaned and immediately frozen in liquid nitrogen and stored at -80 °C until RNA extraction. Plasma also was collected for plasma variable measurements. Statistical analysis was performed using a two way analysis of variance, and significance was accepted at P < 0.05.

**Results:**

A non-significant (P < 0.4, P < 0.79, respectively) increase in ABCA1 and PPAR α genes expression was accompanied by a significant (P < 0.01, P < 0.04, P < 0.04, respectively) reduction in TC, TG, and VLDL-C levels in Crataegus-Pentaegyna groups.

**Conclusions:**

Our findings show that a high intensity treadmill running was able to express ABCA1 and PPAR α in rat heart. Data also possibly indicate that the Crataeguse-Pentaegyna supplementation solely could mimic training effect on the mentioned genes and lipid profiles via different mechanism(s).

## 1. Background

Heart has been recognized as an aerobic tissue with higher metabolic capacity and ATP consumption and it is able to use several substrates such as fatty acids, glycogen, lactate, and ketone bodies for its own energy provision (1-3). Under normal physiological condition, glucose (~30%) and fatty acids (FA) (~70%) are important substrates for cardiac metabolism. Fatty acids come from several sources such as adipose tissue and are transported to the heart after combination with albumin, provision through the breakdown of endogenous cardiac triglyceride (TG) stores, and hydrolysis of TG-rich lipoproteins by lipoprotein lipase (LPL) positioned at the endothelial surface of the coronary lumen (2, 4). An elevated plasma FA induced by fasting or exercise might stimulate related gene expression in the heart tissue. Van der Lee et al. suggested that the exposure of rat ventricular myocyte to long chain fatty acids enhanced the expression of genes involved in fatty acid transport and metabolism (5). Gilde et al. found that the long-chain FA-induced increase in the expression of genes in cardiac muscle cells is mediated not only by PPAR-α, but also by PPAR β/δ (6). It should be noted that the main and effective factor in atherosclerotic cardiovascular disease (CVD) is the accumulation of cholesterol in arterial macrophages and each factor modulating circulation and tissue cholesterol levels has major impacts on initiation, progression, and regression of CVD (7). In this regard, adenosine triphosphate binding cassette transporters (ABC), particularly type A and specially, ABCA1 is recognized as a key element (8) and protector from cardiovascular disease by formation of HDL (7, 9, 10). Although the roles of liver and intestine in plasma HDL and apoA-I secretion are very well known, but the role of heart as an aerobic tissue with high capacity of fatty acids consumption have not been under sufficient attention in relation to the cholesterol regulation and metabolism. Considering the key role of ABCA1 in reverse cholesterol transport process and HDL biogenesis, and the expression of ABCA1 in cardiac muscle in human cell line and animal models (6-12), it is believed that the heart may contribute to this process. Data related to the effect of exercise training on heart ABCA1 expression are very scarce, and to the best of the authors’ knowledge, only one study has focused on the effects of a moderate intensity (25m/min) of treadmill running on ABCA1 mRNA expression of this tissue (9). On the other hand, some suitable medicinal herbs such as Crataegus species (Hawthorn) are introduced as promoter of cardiovascular function and health and are suitable for cardiovascular disease treatment due to having the rich potent antioxidants, and increasing resistance against oxidative stress (13-16). Kwok et al. (17) showed that rats fed with normal diet and high cholesterol diet plus Crataegus pinnatifida Bge (Shan Zha) for four weeks markedly reversed the increased plasma total cholesterol and reduced high density lipoprotein cholesterol induced by HCD. Barros et al. (18) reported that flowers revealed the highest tocopherols and ascorbic acid contents, with the best n-6/n-3 fatty acids ratio. In spite of growing information of the effects of some common cratageus species on cardiovascular health, there is very little information about the effect of Black carategus pentaegyna on cardiovascular function. However, in one study, the antioxidant activity of Crataegus pentaegyna is reported (19). Thus, on the basis of our knowledge up to date, no studies have been done to see the effect of treadmill running at high intensity with or without crataegus pentaegyna extraction on ABCA1 and PPAR-α mRNA expression and selected plasma lipid profiles.

## 2. Objectives

The first purpose of this study was to investigate the effect of high intensity treadmill running program on rat hart tissue ABCA1 and PPAR-α genes expression. The second aim was to see if the administration of aqueous extraction of Crataegus-Pentaegyna could reinforce the possible effect of exercise on ABCA1and PPAR-α expression. The third purpose was to investigate if any possible changes in rat heart tissue ABCA1 and PPAR-α expression are accompanied by any significant changes in plasma lipid profiles.

## 3. Materials and Methods

### 3.1. Animals

All experiments were conducted on the animals according to the policy of Iranian convention for the protection of vertebrate animals for experimental and other scientific purposes; the protocol was approved by the Ethics Committee of the Sciences, University of Mazandaran (UMZ) and Babol University of Medical Sciences (BUMS, Mazandaran, Iran). Twenty Wistar male rats (4-6 weeks old, 140-173 g weight) were acquired from the Pasteur's Institute (Amol, Mazandaran), and maintained in the Central Animal House of the Faculty of Physical Education and Sports Science of UMZ. Five rats were housed in each cage (46-L) for 12-hours: A 12-hour light-dark cycle. Temperature and humidity were maintained at 22°C ± 1.4°C and 50% ± 5%, respectively. Diets (a pellet form) and water were provided ad libitum. Animals were randomly classified into control (n = 10) and training (n = 10) groups. Rats were further divided into saline-control (SC) (n = 5), saline-training (ST) (n = 5), Crataegus-pentaegyna -control (CPC) (n = 5), and Crataegus-Pentaegyna training (CPT) (n = 5). The control (SC and CPC) groups remained sedentary; whereas the training groups underwent a high running exercise program.

### 3.2. Exercise Training Protocol

At first, the animals were familiarized with the rat treadmill apparatus, every day and for five days. The exercise groups were trained for eight weeks on a motor driven treadmill as previously reported elsewhere (20). The rats were submitted to run at 34 m/min for 60 minutes, five d/week (21). The animals were killed 72 hours after the last exercise session. Food not water was removed from the cages three hours before the sacrifices.

### 3.3. Plant Material

The ripped fruit samples of Crataegus-Pentaegyna were collected from Amol and Babol forests in the Mazandaran province of Iran, and washed well. Fruits were dried in oven at 35° C for four days. Plant Material was identified by herbarium collection in department of physical education and sport science, University of Mazandaran, Babolsar, Iran.

### 3.4. Preparation of the Crataegus Extraction

The extraction was prepared according to the Cai et al. (22). Briefly, the whole ripped and dried fruit Crataegus-Pentaegyna were ground to yield fine powder. Water extraction: 5 g of the powdered sample was extracted from 100 mL distilled water at 80° C for 20 min. After cooling, the extract was filtered. Then centrifuged at 3,000 rpm for 17 min and filtered through filter paper. The freshly prepared extracts were cooled and immediately used for the experiments. In last six weeks, all treatments were given in a single daily dose orally using special gavage needles. After training, 500mg/kg liquid extraction of Crataegus-Pentaegyna (23) was orally assigned to the Crataegus-Pentaegyna groups and the same amount of saline was fed to saline groups.

### 3.5. Preparation of the GC/MS Analyses

The whole ripped and dried fruits of Crataegus-Pentaegyna were grounded in house electronic grinder to yield a fine powder, part of which macerated by n-hexane. For 72h at room temperature, they were extracted by soxhlet, and evaporated using a rotary evaporator. Chromatographic analysis was carried out on HP devices, 6890 series GCMS apparatus combined with a mass selective detector. The capillary column used was HP-1MS. Helium was used as carrier gas. The fatty acid components of Crataegus-Pentaegyna extracts were determined using library search soft-ware from Wiley/NBS Registry Mass Spectral Data and in-house “BASER Library of Fatty Acid Constituents”.

### 3.6. Tissue Biopsies

Seventy-two hours after the last training session, rats were anesthetized with intra peritoneal administration of a mixture of ketamine (30– 50mg / kg body weight) and xylazine (3– 5mg / kg body weight). The heart was excised, cleaned, divided into two pieces, washed in ice-cold saline, and immediately frozen in liquid nitrogen and stored at −80° C until RNA extraction. Blood was also collected in EDAT test tubes as anticoagulant, and immediately processed for plasma preparation, during a 15 min centrifugation at 3000 rpm. Plasma was stored at -80C too, for future analysis.

### 3.7. Plasma Lipid Concentrations 

Plasma total Triglyceride (TG) was determined by enzymatic colorimetric method (Pars Azmoun, Tehran, Iran); intra-assay coefficient of variation and sensitivity of the method were 1.53% and 1.23 mg/dL, respectively. Plasma total cholesterol (TC) was determined by enzymatic colorimetric method (Pars Azmoun, Tehran, Iran); intra-assay coefficient of variation and sensitivity of the method were 1.62% and 1.76 mg/dL, respectively. 

### 3.8. RNA, cDNA Synthesis and Real-time PCR

Total RNA was extracted from 30 mg of tissue using RNA purification kits (QIAGEN, Cat.No: 71104). Complementary DNA (cDNA) was extended by using cDNA synthesis kit (QuantiTect Reverse Transcription Kit cDNA synthesis (Qiagen), Cat.No: 205310) according to the manufacturer’s instructions. Real-time quantitative PCR was performed using Quanti Fast SYBR Green PCR Kit (Cat. No. 204052; Qiagen, GmbH, Germany) in using 10µl reaction containing 1µl single-strand cDNA,5µl Master Mix, 1µl of the each forward and reverse primers and 2µl RNase- free water. Expected fragment size and Oligonucleotide primer sequences for ABCA1, PPAR-α and GAPDH genes are listed in Table 1. The PCR was carried out on BIO RAD (C1000 TM Thermal Cycler). Real time PCR system is listed in Table 2. Product specificity was confirmed in the initial experiments by 1.5 % agarose gel electrophoresis and routinely by melting curve analysis.

**Table 1.** Oligonucleotide Primer Sequences and Real-time PCR Amplification Parameters Temperature

Gene	Forward and reverse primer sequences	Annealing temperature	Amplicon size (bp)
**ABCA1**	F: 5׳-acgagattgatgaccgcctc	60 °C	109
	R: 5׳-agcatccaccccactctcttc		
**PPAR-α**	F: 5׳-actcgcaggaaagactagca	60 °C	143
	R: 5׳-agcagtggaagaatcggacc		
**GAP.DH**	F: 5׳-gtgccagcctcgtctcatag	60 °C	199
	R: 5׳-gactgtgccgttgaacttgc		

**Table 2.** Real-time Cycler Conditions

Steps		Time	Temperature
**PCR initial activation step**		5 min	95 ºC
**Two-step cycling**			
**Denaturation**		10 s	95 ºC
**45 Cycle**	**Combined annealing/extension**	30 s	95 ºC
**Melting Curve**		6.5 min	55 to 95 °C

### 3.9. Statistical Analysis

The relative levels of mRNA were analyzed by using a comparative threshold cycle method (CT) (24). The Kolmogorov-Smirnov test was used to determine the normality of distribution, and variables were found to be normally distributed. All results are expressed as means ± SEM. Statistical analysis were performed using a two way analysis of variance. Least significant difference post hoc test was used in the event of a significant (P < 0.05) F ratio. All statistical analysis was performed with SPSS (Version 13; SPSS, Chicago, IL).

## 4. Results

**Figure 1 .**
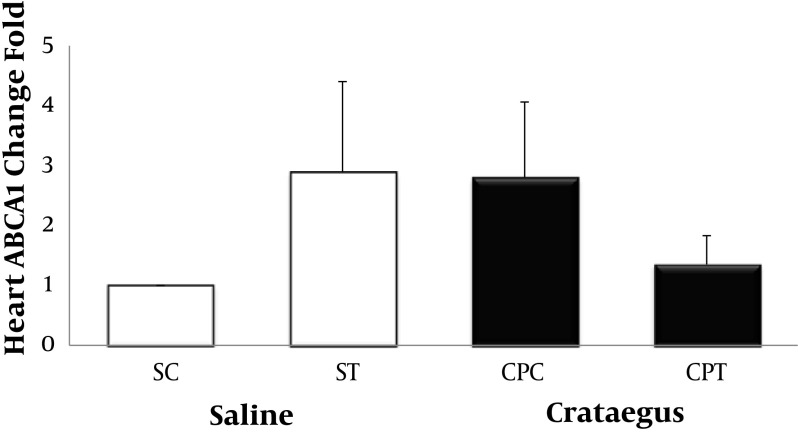
Real-Time PCR of Heart ABCA1 Relative mRNA Expression in Saline- Control (SC), Saline-Training (ST), Crataegus-Pentaegyna -Control (CPC), and Crataegus-Pentaegyna -Training (CPT) Wild-Type male Rats. Wild-type male rats Data expressed as mean ± SEM. Each column is for each group including 5 rats

**Figure 2.**
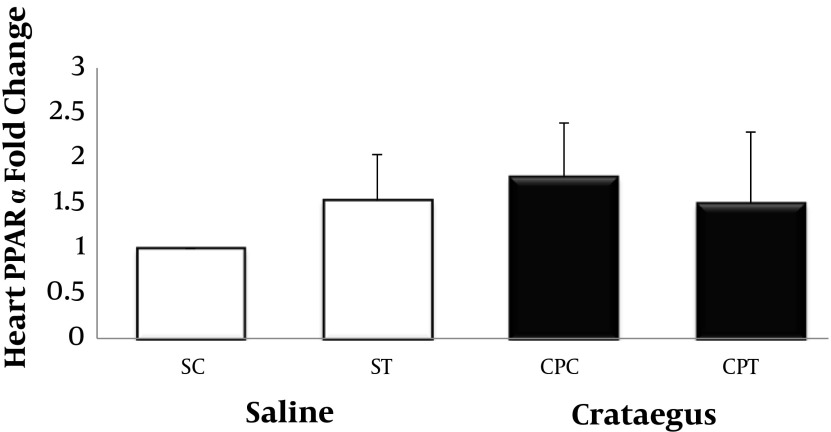
Real-Time PCR of Heart PPAR-α Relative mRNA Expression in Saline- Control (SC), Saline-Training (ST), Crataegus-Pentaegyna -Control (CPC), and Crataegus-Pentaegyna -Training (CPT) Wild-Type male Rats. Wild-type male rats Data expressed as mean ± SEM. Each column is for each group including 5 rats

**Figure 3.**
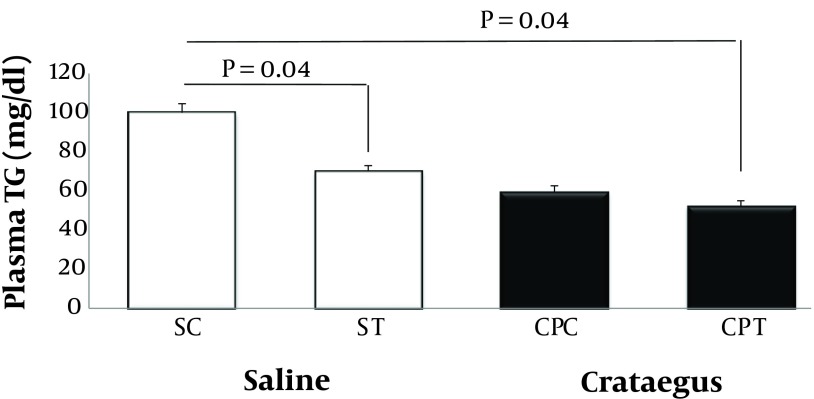
Plasma TG Concentration (mg/dL) in Saline- Control (SC), Saline-Training (ST), Crataegus-Pentaegyna -Control (CPC), and Crataegus-Pentaegyna -Training (CPT) Wild-Type male Rats. Wild-type male rats Data expressed as mean ± SEM. Each column is for each group including 5 rats

**Figure 4.**
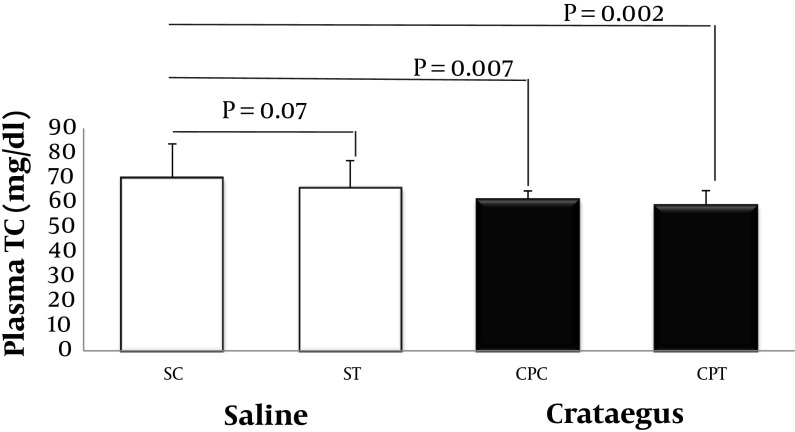
Plasma TC Concentration (mg/dL) in Saline- Control (SC), Saline-Training (ST), Crataegus-Pentaegyna -Control (CPC), and Crataegus-Pentaegyna -Training (CPT) Wild-Type male Rats. Wild-type male rats Data expressed as mean ± SEM. Each column is for each group including 5 rats

**Figure 5 .**
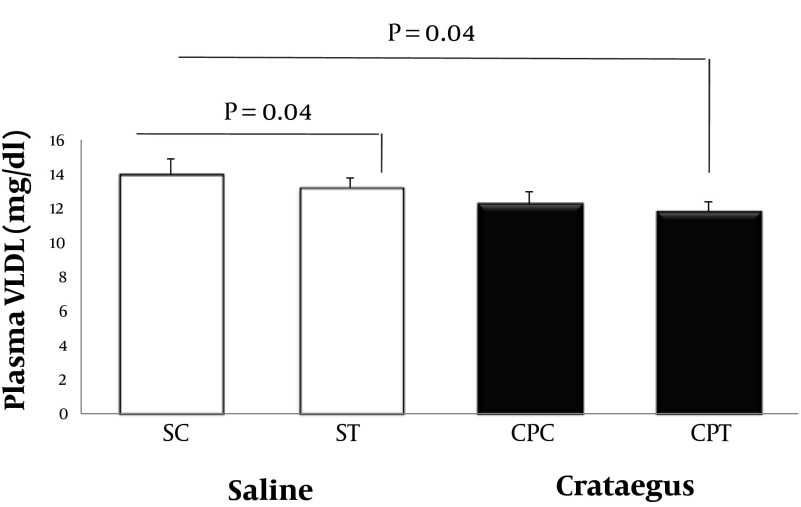
Plasma VLDL Concentration (mg/dL) in Saline- Control (SC), Saline-Training (ST), Crataegus Pentaegyna-Control (CPC), and Crataegus-Pentaegyna -Training (CPT) Wild-Type male Rats. Wild-type male rats Data expressed as mean ± SEM. Each column is for each group including 5 rats

Data analysis revealed that ABCA1 and PPAR-α gene were clearly expressed in rat heart tissue (Figure 1 and Figure 2). Changes in ABCA1 gene expression in response to treadmill running and cratageus supplementation were not significant (Figure 1). However, result indicates that trained cratageus–treated rats had lower ABCA1 gene expression. A significant change was not observed in rat heart PPAR-α after treadmill running or cratageus supplementation between groups (Figure 2). A non-significant plasma TG reduction was observed in cratageus-treated rats compared with saline groups (Figure 3). In this regard, cratageus reduced the plasma TG (P < 0.04) (only for separate P of post hoc test with univariate P that used for cratageus and saline groups) (Figure 3). Plasma TC concentrations were significantly (F = 5.032, P < 0.012) reduced by treadmill running and cratageus supplementation. Using a suitable following post hoc test showed that control-cratageus, trained-Cratageus, and trained saline- treated groups had lower TC concentrations compared with control-saline group (P < 0.007, P < 0.002, and P < 0.03, respectively). In this regard, cratageus reduced the plasma TC (P < 0.007) (Figure 4). No significant difference was found between groups in plasma VLDL concentrations (F = 1.9, P < 0.16.). In this regard, cratageus reduced the plasma VLDL (P < 0.04) (Figure 5) (Table 3).

**Table 3.** Heart ABCA1 and PPAR-α gene expression and Plasma Variables Concentration (mg/dL) in Saline-Control (SC), Saline-Training (ST), Crataegus Pentaegyna-Control (CPC), and Crataegus Pentaegyna-Training (CPT) Wild-type male Rats. Each column is for each group including 5 rats

FGroup Variables	SC	ST	CPC	CPT	F	P value	P value for Interaction
**ABCA1/GAPDH Fold Change**	1	2.89 ± 1.51 ^a^	2.8 ± 1.26	1.35 ± 0.48	0.93	0.44	0.36
**PPAR-α /GAPDH Fold Change**	1	1.53 ± 0.51	1.79 ± 0.60	1.5 ± 0.79	0.35	0.79	0.32
**Plasma Triglyceride, mg/dL**	100 ± 4.47	70 ± 2.91	59.2 ± 3.45	52 ± 2.91	1.95	0.16	0.82
**Plasma Total Cholesterol, mg/dL**	70 ± 13.78	66 ± 10.95	61.4 ± 3.33	59 ± 5.83	5.032	0.012	0.24
**Plasma VLDL, mg/dL**	14 ± 0.89	13.2 ± 0.58	12.28 ± 0.69	11.8 ± 0.58	1.9	0.16	0.82

^a^ Data expressed as Mean ± SD

## 5. Discussion

To our knowledge, this is the first report to demonstrate alterations of male rat heart ABCA1 and PPAR- α relative gene expression in response to a high intensity training and Crataegus extraction regime. In this study, we detected ABCA1 and PPAR-α relative gene expression by Real-time PCR method that is concurrent with previous reports in tissues ABCA1 and PPAR-α gene expression. The major finding of the present study showed no significant increase in ABCA1 and PPAR- α relative gene expression following treadmill running and Crataegus-Pentaegyna extraction regime compared to the control groups. Another finding was a lower TC, TG in Crataegus- Pentaegyna treated rats compared to saline-treated animals. Our observation regarding lack of significant changes in ABCA1 mRNA after eight weeks of high intensity exercise in contrast with previous observations made between rats and humans (9, 25, 26). Ghanbari- Niaki investigated the effect of 12 weeks endurance exercise (intensity: 25m/min, duration: 60min/session and five days a week), that resulted in ABCA1gene expression increase in rat's heart. Plasma apo-A, and pre-β-HDL concentrations, were significantly increased (9). Also, it has been shown that eight weeks of low-intensity exercise (walking), significantly up-regulated ATP-binding cassette transporters A1 (ABCA1) (27). No significant alter of PPAR-alpha in the current study compromise with the results of Tunstall et al. (28) who observed no change in PPAR- α mRNA in humans after nine days of endurance training. This finding is in agreement with Petridou et al. who found no differences between trained and untrained rats in any of the three members of the PPAR family in the liver, skeletal muscle, or adipose tissue after eight weeks ad libitum exercised in cages equipped with wheels (29), but in contrast with those of Russell et al. who observed 2.2-fold increase in PPAR- α mRNA expression after endurance training (30). These conflicting results are most likely explained by the differences in the training duration and intensity. As Russell et al. (30) suggested if a longer training period of at least six weeks at intensities between 60 and %80 of VO_2_max is performed, an increase will be observed in PPAR- α mRNA and protein levels. Hawthorn is well known in phytotherapy for the treatment of many cardiovascular diseases (31), and has been found to decrease the serum levels of cholesterol, LDL-cholesterol, and triglyceride in hypercholesterolemic and atherosclerotic animals (32). The mechanisms by which hawthorn lower LDL cholesterol are not fully understood though a number of hypotheses have been raised. One possibility is the up-regulation of the hepatic LDL receptors. Since some flavonoids such as quercetin have been shown to inhibit the oxidative modification of LDL cholesterol and thus prevent the associated cytotoxicity, it is also possible that hawthorn extract rich in flavonoids exhibits its protective effects via this mechanism (32). Many researchers have shown that Crataegus contain high levels of polyunsaturated fatty acid (PUFA) contents with the best PUFA (saturated fatty acids) SFA ratio, and also the high levels of phenolics (24). Our GC-MS data somehow confirm the previous reports with a little difference, showing that main compositions of the Crataegus-Pentaegyna sample were oleic acid (13.44%), palmitic acid (7.72%), linoleic acid (22.43%) and arashidic acid (0.47%). Several groups have implicated saturated and unsaturated fatty acids as natural ligands for PPAR-α. Natural PPAR-α ligands in human serum include palmitic acid, oleic acid, linoleic acid, and arachidonic acid. Notably, PPAR-α is the only PPAR subtype that binds to a wide range of saturated fatty (33). PPAR- α is highly expressed in tissues (liver, kidney, heart, muscle, adipose tissue) with high rates of fatty acid catabolism, and PPAR- α activators increase ‘reverse cholesterol transport’ by accelerating the efflux of cholesterol from peripheral cells and increasing its uptake into liver through a pathway involving increased vascular expression of the HDL-c receptors, ATP-binding cassette transporter-I (ABC-I) and scavenger receptor class-B type-I (SR-BI) (34). Thus, by activating ABCA1, it is possible to inhibit the progression of premature atherosclerosis. In conclusion, the present study showed that high intensity exercise influences genes probably through different mechanisms. Also it is demonstrated that the Crataeguse-Pentaegyna without training is able to increase gene expression and decrease the risk factors. Further investigation of the effects of exercise training at low or moderate intensity whit supplementation of Crataegus fruits, flowers and leaves extraction on the gene expression of ABC family and PPAR isoforms in heart muscle is warranted.
